# Modulation of Melanocyte in Melasma Patients After Picosecond Laser Treatment

**DOI:** 10.1111/jocd.16569

**Published:** 2024-09-16

**Authors:** Ching‐Li Chen, Chih‐Chiang Chen, Feng‐Ling Tsai, I‐Ling Chen, Ling Huang, Yu‐Chun Yen, Chian‐Yaw Hwang

**Affiliations:** ^1^ Department of Dermatology Taipei Veterans General Hospital Taipei Taiwan; ^2^ Faculty of Medicine, School of Medicine National Yang Ming Chiao Tung University Taipei Taiwan; ^3^ Department of Dermatology National Yang Ming Chiao Tung University Taipei Taiwan; ^4^ Institute of Clinical Medicine National Yang Ming Chiao Tung University Taipei Taiwan; ^5^ Apollo Medical Optics, Ltd Taipei Taiwan; ^6^ Yawen Dermatology New Taipei City Taiwan

**Keywords:** cosmetic dermatology, dermatology, laser treatment, melasma, Nd:YAG laser, noninvasive testing

## Abstract

**Background:**

Melasma is a therapeutically challenging hyperpigmented skin condition. Currently, there is a lack of in vivo observation regarding changes in melanin and dendritic melanocytes after laser treatment.

**Objective:**

To investigate alterations in melanin and melanocytes in melasma before and after laser treatment using optical coherence tomography (OCT).

**Methods:**

Eight female melasma patients were enrolled in Taiwan. Based on the baseline OCT scans, the patients were categorized into either epidermal‐type or mixed‐type melasma and were assigned different treatment protocols accordingly. Sequential OCT images were collected from melasma lesions and normal skin at baseline, Week 4 and Week 8.

**Results:**

After 8 weeks of laser treatment, the mean Melasma Area Severity Index (MASI) score improved from 10.92 to 6.30. Results from OCT showed no significant changes in the normalized density, area, or intensity of melanin in both lesional and normal skin. At baseline, the mean length of dendritic melanocytes in the affected skin was 15% longer than those in normal skin; at Week 8, the mean length of lesional dendritic melanocytes became the same as those in normal skin. Additionally, the mean width of dendritic melanocytes decreased from being 4% wider to only 2% wider than those in normal skin.

**Conclusion:**

After 8 weeks of treatment, an improvement of MASI score was noted, mainly attributable to a reduction in lesional area. OCT showed no notable change regarding melanin, but a decrease in length and width of dendritic melanocytes was noted in the lesional skin of melasma patients.

## 1 Introduction

Melasma is an acquired cutaneous disease characterized by irregular, hyperpigmented patches located symmetrically on the face. It predominantly affects individuals residing in tropical regions and with Fitzpatrick skin types III–IV [1]. The pathophysiology of melasma is intricate, and various triggering factors have been observed, including exposure to ultraviolet radiation, use of cosmetics, medications, oral contraception, and hormone replacement therapy [2, 3].

The treatment of melasma is challenging and often needs a long‐term treatment with a multifaceted approach. Melasma can be classified based on the depth of skin involvement into epidermal, dermal, or mixed types [4]. The determination of the subtypes is crucial as it influences the response to therapeutic interventions. Accurate subtyping of melasma can aid in prognosis prediction and the formulation of precise treatment plans. Conventionally, classification was primarily carried out by experienced clinicians through visual examination and Wood's lamp illumination. Wood's lamp can help distinguish between these entities because it enhances the contrast of epidermal pigmentation while diminishing the contrast of dermal pigmentation. However, this assessment does not always align with histological findings [4]. Recently, many noninvasive modalities have been developed to examine the detailed architecture of skin. For example, reflectance confocal microscopy (RCM) and optical coherence tomography (OCT) both provide real‐time, nearly histological‐level skin visualization [5, 6]. Nevertheless, there is still a lack of discussion on the use of these new techniques to evaluate the effectiveness of treatments for pigmented skin conditions. In the current study, we employed OCT to investigate alterations in melanin and melanocytes in melasma before and after laser treatment.

## 2 Materials and Methods

### 2.1 Study Group

This observational study enrolled eight Asian women diagnosed with melasma in Taiwan. The exclusion criteria included pregnancy, breastfeeding, any concurrent cutaneous or systemic disease requiring treatment with isotretinoin, steroids, or photosensitizing drugs, and the use of lightening treatments. Informed consent was obtained from all participants. The patients were assigned to different treatment protocol based on the baseline OCT findings. Patients with epidermal‐type melasma underwent treatment with a 532 nm picosecond laser (Picocare; Wontech Co., Ltd., Daejeon, Korea) with 7 mm spot size and a fluence of 0.2 J/cm^2^; patients with dermal and mixed‐type melasma underwent treatment with a 1064 nm picosecond laser (Picocare; Wontech Co., Ltd., Daejeon, Korea) with 7 mm spot size and a fluence of 0.7 J/cm^2^. A microlens array was applied during the treatment of both groups. Each patient received two treatment sessions, with each session spaced 28 days apart.

### 2.2 Clinical Assessment and Therapeutic Procedure

The clinical intensity and size of hyperpigmented lesions were evaluated using Melasma Area Severity Index (MASI). The MASI score is calculated by assessment of three factors: area (*A*) of involvement, darkness (*D*), and homogeneity (*H*), yielding a total score ranging from 0 to 48 points [7, 8]. MASI score was performed at each time point by two trained raters.

OCT examination was performed at baseline (W0), fourth week (W4), and Weeks (W8), respectively. “ApolloVue” OCT Image System, Model: S100 (Apollo Medical Optics, Ltd., Taipei, Taiwan) was used in this study to provide real‐time full‐field cellular resolution images. The system provides both cross‐sectional and en face (horizontal section) imaging modes. Five melasma lesional fields of views (FOVs) and 10 non‐lesional FOVs were obtained from the left cheeks of each patient. A computer‐aided detection system was utilized for melanin detection and quantification. En face images were acquired continuously from stratum corneum to reticular dermis. To quantify melanin levels, we analyzed the en face image located 15 μm above the dermoepidermal junction utilizing a computer‐aided detection (CADe) system [5]. A previous pilot study demonstrated that this technique provides cellular resolution with a strong correlation to histological findings concerning various skin disorders in the epidermis and upper dermis [9]. Following established criteria, all targets with a diameter larger than 0.5 μm and a brightness level exceeding 153 grayscale units were identified as melanin [5, 10]. Moreover, the CADe system is employed to evaluate the quantity of activated melanocytes in various parameters, including total area, mean size, maximum size, length, width, and mean intensity. Given the significant interpersonal variability in baseline melanin distribution, normalization was conducted to observe the treatment effect accurately. Collagen quantification was conducted using cross‐sectional images. Contrast‐limited adaptive histogram equalization was employed to augment local contrast and accentuate the characteristics of collagen, which exhibit stronger intensity compared to the surrounding signal [11]. Subsequently, collagen was detected utilizing the Frangi method, incorporating specified criteria for thickness sensitivity and brightness level [12]. The obtained results underwent a refinement process to mitigate the interference caused by oblique lighting, small areas, and variations in image depth.

### 2.3 Data Analysis

Data analyses were performed using Microsoft Excel 365 (Microsoft Corporation, Washington, United States). Quantitative data are presented as the mean with standard deviation. In the comparison of treatment effects, normalization was implemented to mitigate interpersonal differences. Specifically, the normalization process entailed dividing data from lesional skin by that of normal skin.

## 3 Results

### 3.1 Patient Characteristics and MASI Score

The demographic characteristics of the patients are shown in Table 1. Upon baseline OCT analysis, six patients were diagnosed with epidermal‐type melasma and received treatment with 532 nm picosecond laser; the other two patients were diagnosed with mixed‐type melasma and underwent treatment with 1064 nm picosecond laser. At the initial assessment, the mean MASI score for all patients was 10.92 ± 6.22. Regarding the individual components of the MASI score at baseline, the mean sum of area of involvement was 10.69 ± 3.65, darkness was 6.44 ± 1.99, and homogeneity was 5.94 ± 1.59. Following laser treatment, the mean MASI score at W4 was 7.79 ± 4.12 and further decreased to 6.30 ± 3.74 at W8. Upon closer examination of the three components of the MASI score, it was observed that all domains exhibited a decreasing trend, with the involved area (“A” score) showing the most significant change.

**TABLE 1:** Patient characteristics.

Variate	Mean ± SD
Age (year)	48 ± 4.63
Gender	
Male	0 (0%)
Female	8 (100%)
Fitzpatrick skin type	
III	8 (100%)
Type of melasma	
Epidermal type	6 (75%)
Mixed type	2 (25%)
MASI score (W0)	
Total	10.92 ± 6.22
Area	10.69 ± 3.65
Darkness	6.44 ± 1.99
Homogeneity	5.94 ± 1.59
MASI score (W8)	
Total	6.30 ± 3.74^a^
Area	7.06 ± 2.87^a^
Darkness	4.63 ± 1.30
Homogeneity	4.19 ± 0.84

Abbreviations: SD, Standard deviation; W0, Week 0 (baseline); W8, Week 8.

^a^ The data achieved statistical significance when compared to the baseline (W0).

### 3.2 Melanin and Melanocyte

The findings regarding melanin and melanocyte detection via OCT are summarized in Table 2. At the baseline assessment, the mean density of total melanin over normal skin was 7.66% ± 0.89%, with a mean area of 19 226.30 ± 2278.6 μm^2^. When compared to the baseline, no significant changes in the normalized density, area, and intensity of melanin were observed at W8. These findings were consistent for both lesional and normal skin.

**TABLE 2:** Change in melanin and dendritic melanocytes of normal and lesional skin detected by optical coherence tomography.

	Normal skin			Lesional skin (normalized^a^)		
	W0 (mean ± SD)	W8 (mean ± SD)	*p*	W0 (mean ± SD)	W8 (mean ± SD)	*p*
Melanin						
Area (μm^2^)	19 226.30 ± 2278.60	18 577.52 ± 2543.24	0.51	0.94 ± 0.14	1.01 ± 0.05	0.25
Density (%)	7.66 ± 0.89	7.37 ± 0.77	0.42	0.95 ± 0.12	1.02 ± 0.07	0.22
Intensity (%)	50.28 ± 1.17	50.12 ± 1.6	0.60	0.99 ± 0.02	0.99 ± 0.02	0.45
Dendritic melanocyte						
Size (μm^2^)	41.4 ± 14.38	42.95 ± 12.00	0.55	1.23 ± 0.29	1.14 ± 0.31	0.05
Length (μm)	26.66 ± 5.47	29.01 ± 3.02	0.11	1.15 ± 0.15	1.00 ± 0.18	<0.01
Width (μm)	1.59 ± 0.30	1.65 ± 0.14	0.47	1.04 ± 0.15	1.02 ± 0.14	0.04
Intensity (%)	50.4 ± 11.12	50.3 ± 5.31	0.97	1.11 ± 0.20	1.08 ± 0.19	0.45

Abbreviations: SD, standard deviation; W0, Week 0 (baseline); W8, Week 8.

^a^ Normalization was executed through the division of lesional skin data by that of normal skin.

### 3.3 Dendritic Cells

At W0, the mean length and width of dendritic melanocytes in lesional skin were 15% longer and 4% wider than those in normal skin, respectively. After two laser treatment sessions, the mean length of dendritic cells decreased to the same length as normal skin, and the width reduced to only 2% wider than normal skin (Table 3).

**TABLE 3:** Change in collagen of normal and lesional skin detected by optical coherence tomography.

	Density (%)		Length (μm)	
	Mean + SD	*p* (vs W0)	Mean + SD	*p* (vs W0)
Normal skin				
W0	29.09 + 1.75	—	62.17 + 5.95	—
W8	29.28 + 1.36	0.76	63.53 + 5.21	0.55
Lesional skin				
W0	27.86 + 2.67	—	59.27 + 7.41	—
W8	29.85 + 1.27	0.07	65.47 + 5.38	0.09

Abbreviations: SD, standard deviation; W0, Week 0 (baseline); W8, Week 8.

### 3.4 Collagen

At W0, the mean density of detected collagen was 29.09% ± 1.75% at normal skin and 27.86% ± 2.67% at lesional skin. By W4, the mean density of detected collagen in lesional skin increased to 30.49% ± 3.13%, while the density of collagen in normal skin was 29.49% ± 1.53%. At W8, the mean density of collagen was 29.28% ± 1.36% in normal skin and 29.85% ± 1.27% in lesional skin. After dividing the patients by different lesion types, the analysis revealed that collagen density in lesional skin in the epidermal type/532 nm‐laser group was 27.34% ± 2.81% at W0 and increased to 29.56% ± 1.21% at W8. In comparison, the mixed type/1064 nm‐laser group showed collagen density of 29.42% ± 1.94% at W0, which became 30.71% ± 1.41% at W8.

## 4 Discussion

The study found an improvement in MASI score after 8 weeks of picosecond laser treatment. OCT of lesional skin identified a reduction in length and width of the activated melanocytes, while the melanin density, area, and intensity remained relatively stationary.

Determining the depth of involvement is crucial in melasma treatment planning. In this study, we used 1064 nm picosecond laser for patients with mixed‐type melasma and 532 nm picosecond laser for those with epidermal type. Theoretically, the shorter laser pulse durations in picosecond laser make it more efficient at pigment removal with less thermal damage to surrounding tissue [13]. The treatment effects of a fractional picosecond 1064 nm laser in treating melasma have been demonstrated in multiple studies [14, 15]. On the other hand, the efficacy of 532 nm picosecond laser on melasma was less investigated. Compared to 1064 nm, 532 nm is more selectively absorbed by melanin and has shallower penetration depth, potentially causing less damage to surrounding blood vessels. Therefore, it is a better option for treating epidermal‐type melasma. In our study, six out of the eight patients were identified as having epidermal type melasma. Overall, picosecond laser treatment resulted in a notable improvement in mean MASI score (W0: 10.92 ± 6.22 vs. W8: 6.30 ± 3.74).

The MASI score is calculated by assessment of three factors: area (*A*) of involvement, darkness (*D*), and homogeneity (*H*), yielding a total score ranging from 0 to 48 points [7, 8]. Our study highlighted that the most significant improvement in the MASI score was attributed to the “area” component. This indicates that the improvement in melasma may be more closely linked to a reduction in the size of the affected area rather than changes in skin darkness at W8.

Despite marked clinical improvement, OCT did not detect significant changes in melanin density, area, or intensity at W8. This observation aligns with the finding that the improvement in MASI score at W8 is primarily due to a reduction in the size of the affected area rather than changes in skin darkness. However, our study revealed an opposite response of dendritic melanocytes in lesional and normal skin following laser treatment. In lesional skin, both the relative width and length of dendritic melanocytes decreased, whereas in normal skin, the data either remained stable or slightly increased. Several investigations into the response of melanocytes to light exposure have been conducted in the past, but the results have been controversial. It has been reported that lesional and non‐lesional skin display different reaction to light exposure. Gao et al. enrolled 25 melasma patients and used RCM to detect the change in melanin and dendritic cells after 1064 nm Nd:YAG laser treatment [16]. They observed dendritic cells in lesional skin in 8 out of the 25 patients at baseline and in all 25 patients after 10 sessions of laser treatment. Conversely, there were no discernible dendritic cells in the non‐lesional skin of all 25 patients, both before and after laser treatment [16]. Wang et al. found that shortly after exposure to ultraviolet (UV) light, confetti melanin increased in perilesional skin but not in melasma lesions [10]. They proposed that the melanocytes of melasma lesion have lost their adaptability in response to UV irradiation, and the shedding of melanosome‐rich packages persistently plateaus [10]. In contrast, melanocytes in perilesional skin still respond to UV irradiation by actively transporting melanosome‐rich packages, and this contributes to the worsening of melasma after UV exposure [10]. In our study, we observed a decrease in relative length and width of lesional dendritic melanocytes at 8 weeks following laser treatment. Our findings suggest that laser treatment may modulate dendritic melanocytes in melasma patients. It is important to note that the aforementioned studies were conducted at different follow‐up time frames and utilized diverse light sources. To gain a more comprehensive understanding of the dynamics of melanin and melanocytes after light exposure, observing melanocytes using lasers of different wavelengths at various time points is necessary.

In our study, we observed an increase in collagen levels in lesional skin. The creation of laser‐induced thermal breakdown (LITB) has been observed in both 532 and 1064 nm picosecond lasers with a microlens array [17]. LITB induces the formation of vacuoles in the tissue, triggering the release of cytokines upon damaging keratinocytes. These cytokines facilitate dermal collagenesis, wound healing, and dermal remodeling [18].

Our study has several limitations. First, the sample size is small; a larger sample size would be necessary to facilitate robust statistical analysis. Besides, due to the relatively short follow‐up period, our findings may only capture the initial changes rather than the entire course of melasma treatment. Third, our protocol exclusively assessed melanin within 15 μm above dermoepidermal junction, so pigment changes in dermis was not taken into account. Additionally, the correlation between OCT images and histological findings regarding melanin, dendritic melanocytes, and collagen requires further validation.

In summary, our study utilized OCT to investigate the treatment response regarding melanin, dendritic melanocytes, and collagen in patients with melasma. Following an 8‐week treatment period, we observed an improvement in MASI score, primarily attributed to a reduction in the lesional area. In terms of OCT findings, we noted a decrease in both the length and width of dendritic melanocytes in the lesional skin, while melanin density and intensity remained relatively unchanged. These findings suggests that clinical improvement at W8 in melasma may be attributed more to a decrease in the affected area rather than a reduction in melanin density. Further research with a larger sample size and an extended follow‐up period is necessary to provide a longitudinal observation of melanin and melanocyte following laser treatment.

## Disclosure

Dr. Chian‐Yaw Hwang holds positions as a board member and shareholder at Apollo Medical Optics. However, this research did not receive any funding or grant from the company. The patients in this study have given written informed consent to publication of their case details.

## Conflicts of Interest

The authors declare no conflicts of interest.

## Data Availability

The data that support the findings of this study are available on request from the corresponding author. The data are not publicly available due to privacy or ethical restrictions.
